# The importance of paraoxonase 1 activity in chronic kidney disease

**DOI:** 10.1080/0886022X.2024.2376930

**Published:** 2024-07-10

**Authors:** Elisabeth C. Samouilidou, Augustina Liaouri, Vassilis Kostopoulos, Dimitris Nikas, Eirini Grapsa

**Affiliations:** a Department of Biochemistry, “Alexandra” Hospital, Athens, Greece; b Renal Unit, “Aretaeio” Hospital, Athens, Greece

**Keywords:** Paraoxonase 1 activity, antioxidants, chronic kidney disease, hemodialysis, cardiovascular disease

## Abstract

Paraoxonase 1 (PON1) is one of the most significant antioxidative enzymes associated with high-density lipoprotein (HDL). It has been proved that is involved in the pathogenesis of many diseases including chronic kidney disease (CKD). The association between PON1 and CKD seems to be mutual, such that the disease produces a significant decrease in PON1 activity levels, while the genetics of PON1 may affect the risk of susceptibility to CKD. Recent studies reveal that the decrease in serum PON1 activity observed in non-dialyzed and dialyzed CKD patients as well as in renal transplant (RT) patients is linked to an increased vulnerability to atherosclerosis. We intend to summarize current literature concerning PON1 activity in CKD, highlighting on the main determinants of PON1 activity, its association with oxidative stress, the impact of its genetic polymorphism on the disease development, the effect of drugs and nutritional state. Furthermore, evidence supporting the implication of reduced PON1 activity in the incident of cardiovascular disease in CKD patients, is also examined. It appears that despite the lack of standardization of PON1 activity measurement, PON1 remains a valuable biomarker for the researchers through the last decades, which contributes to the assessment of the antioxidant status having prognostic benefit on adverse clinical outcomes at various stages and etiologies of kidney disease.

## 1. Introduction

Chronic kidney disease (CKD) is defined as abnormalities of kidney structure or function, present for a minimum of 3 months, with implications for health [1]. The global prevalence of CKD is estimated to be 14% and has resulted in 1.43 million deaths worldwide. Patients with CKD have frequently cardiometabolic disorders, such as hypertension, diabetes and dyslipidemia, which can contribute to oxidative stress [1,2]. Antioxidant enzymes as paraoxonase 1 (PON1), play a key role in tackling oxidative stress and facilitating the antiatherogenic action of high- density (HDL) [3,4].

PON1 is a calcium-dependent aryldialkyl phosphatase with a molecular mass of 45 KDa, whose polypeptide chain consists of 354 amino acids. It is a component of HDL assembly, associated with apolipoprotein AI (apo A-I) and clusterin (apolipoprotein J) [3–5]. The PON1 gene is a member of paraoxonase gene family, which includes also PON2 and PON3 genes, located next to each other on the long arm of human chromosome 7 [6]. PON2 protein is a ubiquitous intracellular protein, localized mainly in mitochondria, endoplasmic reticulum and plasma membrane, that does not associate with HDL particles in circulation. PON1, as PON3, is produced predominantly in the liver, secreted in the circulation and delivered to target tissues through binding with HDL [6]. PON1 is capable of hydrolyzing a large variety of substrates, having therefore multiple activities, which are suggested to be connected with different physiological functions. It can hydrolyze organophosphate compounds such as paraoxon (paraoxonase activity), which explains its ability to hydrolyze organophosphorus insecticides. It can also hydrolyze aromatic carboxylic acid esters, such as phenyl acetate (arylesterase activity). Most importantly, it can hydrolyze homocysteine-thiolactone (homocysteine-thiolactonase activity), and this is claimed to be linked with the beneficial action of PON1 in the prevention of protein honocysteinylation, cell damage and atherosclerosis [7,8]. On the other hand, PON1 hydrolyzes phospholipid hydroperoxides (peroxidase activity). This seems to be of great importance in the detoxification of oxidative stress mediators and possibly explain the antioxidant and anti-inflammatory potential of the enzyme [3,4]. Studies corroborate that different catalytic sites and conformations of PON1 structure are responsible for different activities [6,9]. The native activity of PON1 is attributed to homocysteine-thiolactonase (lactonase), while the activities mostly measured in the laboratory for kidney diseases are those of paraoxonase and arylesterase [10,11]. Immunohistochemical evidence for the expression of PON1 in rat kidney tissue show that PON1 is present in the proximal tubule, a site which is particularly susceptible to chemical insult [12]. It is thus possible that apart from its antioxidant, anti-inflammatory and antiatherogenic functions, PON1 in kidney could also contribute in protecting against the toxic effect of xenobiotics, that are both filtered through the glomeruli or diffuse back from urine filtrate to the serum across the tubular cells.

PON1 is an HDL-associated protein that has primarily the ability to hydrolyze oxidized LDL-cholesterol. The association of PON1 with HDL ensures many of its anti-inflammatory and anti-atherogenic properties [3,4]. The most essential properties ascribed to PON1 are the protection of LDL from oxidation, the restriction of plasma oxidized LDL, the stimulation of HDL-mediated cholesterol efflux from macrophages, the activation of reverse cholesterol transport, the inhibition of foam cell formation, the suppression of monocyte chemotactic protein-1 (MCP-1) production and the detoxification of homocysteine thiolactone [13,14]. In CKD, changes in lipoprotein metabolism and the development of dyslipidemia induce abnormalities in all lipoprotein classes, with variations depending on the degree of renal impairment, the etiology of primary disease and the type of dialysis for patients undergoing renal replacement therapy. Dyslipidemia may affect HDL-dependent antioxidant enzymes, including PON1 [15,16]. In this review, we summarize current evidence regarding PON1, emphasizing the importance of PON1 activity in chronic kidney disease.

## 2. The alterations of PON1 activity in chronic kidney disease

### 2.1. Diadetic nephropathy

Diabetic nephropathy (DN) is one of the common complications of diabetes, leading to end-stage renal failure. Initial phases of DN are characterized by microalbuminuria, which is present even when even there is not yet evidence of abnormal glomerular filtration, while on progression, microalbuminuria advances to an overt proteinuria (macroalbuminuria) [17]. Ikeda et al. firstly aimed to estimate serum PON activity in patients with non-insulin dependent diabetes (NIDDM) and its relationship to diabetic complications, including nephropathy, retinopathy, neuropathy and macroangiopathy [18]. They discovered that PON activity in NIDDM patients was significantly lower than in controls and that the subgroup of patients having retinopathy or overt proteinuria had significantly decreased levels of PON activity compared to the respective subgroup without retinopathy or proteinuria. Logistic analysis revealed that serum PON activity was one of the significant factors of retinopathy, however, PON activity was not a significant factor for the risk of nephropathy [18]. Zhou et al. investigated whether serum level of advanced glycated end products (AGEs), which are enhanced by hyperglycemia and by oxidative stress, could influence the antioxidant capacity of HDL in type 2 diabetic patients with and without nephropathy [19]. Using a functional assay, they observed that PON1 in HDL isolated from diabetic patients with microalbuminuria or proteinuria was decreased compared to PON1 from normoalbuminuric diabetic patients, and was inversely correlated to AGEs levels. This led to the suggestion that increased AGEs in diabetic subjects might result in a loss of PON1 activity, associated with impaired antioxidant ability of HDL. Notably, in other reports, although PON1 activity was decreased in diabetic patients compared to healthy subjects [20–22], it did not differ between diabetic patients with or without nephropathy [21,22]. In diabetic patients with nephropathy, PON1 activity was negatively correlated with the oxidative stress marker malondialdehyde (MDA) [23], but not with the disease duration and glycemic status [22]. It should be pointed out that hemodialysis was proved to exacerbate PON1 activity in diabetic patients compared with non-diabetic HD patients [24]. This was not conspicuous in the case of PD, where no difference was observed regarding the PON1 activity between patients with or without diabetes [24].

### 2.2. Hypertensive nephropathy

Hypertension is a common condition observed in CKD that can lead to end-stage renal disease [25]. Although dyslipidemia seems to be associated with the risk of development of hypertension [26], and affects PON1 activity in CKD [15,16], there are sparse data on PON1 activity levels in patients with hypertensive nephropathy. In a study with adolescent patients with essential, obesity- induced and uremic hypertension, it was found that PON activity was significantly lower in the uremic hypertensive group than in the controls [27]. Nevertheless, no significant correlation was observed between PON1 activity nor PON1 genotypes and the distribution of biochemical parameters pertinent to endothelial dysfunction (end-products of nitric oxide) and oxidative stress (oxidized and reduced glutathione, lipid peroxides and malondialdehyde equivalents) in hypertensive groups [27]. In a larger- scaled cohort study with participants without preexisting hypertension in the Prevention of Renal and Vascular End-stage Disease (PREVENT), followed-up for 10.7 years, PON1 arylesterase activity was not associated with future risk of hypertension, even though there is an inverse and independent correlation between hypertension risk and HDL-C, not modified by PON1 [28].

### 2.3. Renal replacement therapy and renal transplantation

**Figure 1.**
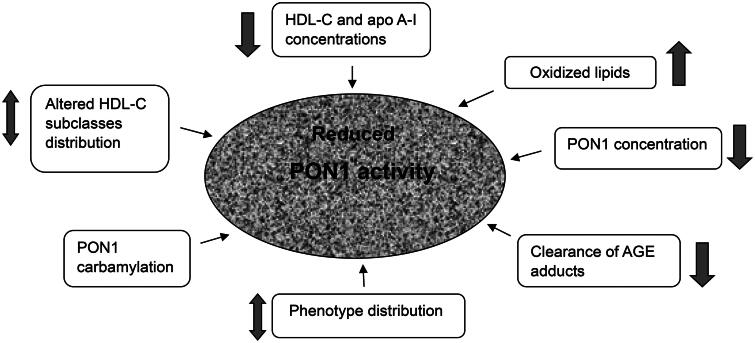
Potential causes of reduced PON1 activity levels in chronic kidney disease (CKD).

Schiavon et al. firstly studied serum paraoxonase activity in uremic patients under long-term hemodialysis (HD) treatment. They found that it was significantly lower compared with the activity levels in normal controls (NC) [29]. Also, in the patient group the effect of NaCl on paraoxonase activation was more pronounced than in NC group. According to previous observations, a higher stimulation of paraoxonase by NaCl suggested a higher frequency of paraoxonase B allozyme (more responsive to NaCl), linked to the presence of a single amino acid polymorphism (Gln-Arg 192) of the paraoxoase protein [30]. Thus, it was suggested that altered paraoxonase phenotype distribution could be a possible explanation of reduced activity levels in HD patients (Figure 1). Although it was recognized in this study that the effect of NaCl on the enzyme activity was only an indirect approach to define the allozyme, and the sample size of HD patients was rather small, it was assumed that the higher frequency of B allozyme among HD patients may be an adjunctive index of altered lipoprotein metabolism with a significant impact on atherosclerosis. Dantoine et al. conducted research in non-end-stage chronic renal failure patients on HD, peritoneal dialysis (PD) and in renal transplant (RT) patients, measuring paraoxonase activity by three substrates (phenyl acetate, paraoxon and 4-nitrophenyl acetate) [31]. It was found that whatever the substrate, paraoxonase was markedly lower in patients suffering from CKD than in control subjects, especially in HD and PD groups. In RT patients, paraoxonase did not differ significantly from the controls. Therefore, it was concluded that the decrease of paraoxonase activity could be an important factor of premature vascular aging in dialyzed patients and that renal transplantation appears to restore paraoxonase activity. These findings were in line with the results obtained by Paragh et al. [32], who compared an HD group with a RT group of patients. Herein, to exclude that the reduction in PON activity in HD patients was due to decreased serum HDL levels, the standardized PON activity for HDL concentrations (PON/HDL ratio) was further used to demonstrate that it was also significantly reduced in HD patients compared to the RT patients. The phenotype distribution of PON, evaluated by the ratio of the hydrolysis of paraoxon in the presence of NaCl (salt-stimulated PON) to the hydrolysis of phenyl acetate, revealed that the low- activity AA phenotype and the intermediate-activity AB phenotype accounted for more than 90% of the patients and controls [32]. In relative opposition to the former results [29], the high-activity BB phenotype was the lowest in the uremic patients. However, the absence of significant phenotypic difference between groups, supported the assumption that the decrease in serum PON activity in patients could not be explained on the basis of their altered phenotypic distribution, as postulated also in other population groups [33]. Henning et al. extended the findings about paraoxonase and HD, showing that dialysis vintage (i.e., time on dialysis), induced a significant reduction of PON ratio (calculated from 4-nitrophenyl acetate- derived activity divided by phenylacetate-derived activity) [34]. Patients who had been treated with HD for more than 60 months displayed lower PON ratio compared to patients treated for less than 12 months, a fact that seems to be related to a shift of phenotype. Gugliucci et al. examined the accumulation of advanced glycation end (AGE) products and advanced oxidation protein products in plasma as another possible cause for the decline in PON1 activity in HD patients [35]. They showed that PON1 activity increased after hemodialysis, with values ranging from 4 to 40% of those before dialysis. This increase displayed strong positive correlation with the rates in which creatinine and urea are cleared, and with the efficiency of low molecular AGEs clearance. Moreover, serum ultrafiltrates of HD patients inhibited PON1 activity *in vitro* in a concentration and time-dependent manner. The above observations were confirmed in a subsequent study based on the homocysteine-thiolactonase activity of PON1 [36], which is found to be linked to anti-atherogenic properties [7,11,37]. However, its determination *via* the substrate TBBL (5-thiobutyl butyrolactone) and not *via* its natural substrate homocysteine-thiolactone, was a limitation mentioned in this study.

Miljkovic et al. examined the possible alterations in the distribution of PON1 activity on HLD2 and HDL3 subclasses, separated from HDL particles derived from end-stage renal disease (ESRD) patients on HD, and from non-dialyzed patients [38]. The data revealed that ESRD patients have lower PON1 concentration and paraoxonase activity than CKD patients. PON1 concentration and its activities on HDL2 and HDL3 subclasses were significantly decreased in patients compared to controls, and that the proportion of PON1 activity on smaller-sized proatherogenic HDL3_a_ and HDL3_b_ particles was significantly increased, probably inferring that the risk for the development of atherosclerosis in CKD patients was elevated. Additionally, the effect of oxidative stress and lipid peroxidation on the decrease of PON1 activity was further investigated in several reports concerning HD and PD [35,39–41]. It was observed that paraoxonase and arylesterase activities were negatively correlated with serum lipid hydroperoxides levels in HD patients [39], and CRF patients on conservative management [40]. In the latter case, it was proved that the decrease in paraoxonase activity, more pronounced in HD than in CRF patients, correlated positively with protein thiols, which are known to confer to protection against LDL oxidation [42]. In patients on continuous ambulatory peritoneal dialysis (CAPD), decreased serum PON1 activity was significantly correlated with several factors influencing nitric oxide cycle, such as nitric oxide synthetase (NOS), asymmetric dimethylarginine (ADMA), ceruloplasmin (CP), oxidized LDL, thiobarbituric acid-reactive substances (TBARS), and malondialdehyde (MDA) [41]. Nevertheless, on multiple logistic regression analysis, risk factors associated with CAPD did not include PON1 activity levels.

### 2.4. Non-dialysis chronic kidney disease

In non-dialyzed CKD patients, evidence about PON1 activity seems to be controversial, not only due to diverse substrates employed to measure PON1 activity, but also due to different stages of CKD selected in each study and to dissimilarities in the percentage of subjects under statin therapy. In patients with low glomerular filtration rates (GFR) (stage 3 or higher), it was found that PON1 activity (as well as HDL-C levels) did not differ from NC, although was significantly correlated with lipid hydroperoxide levels [43]. In a more recent study [44], it was observed that neither PON1 activity nor the HDL-associated enzymes myeloperoxidase (MPO) and lecithin-cholesterol acyltransferase (LCAT) differed in stages 3a 3b and 4 of CKD. In contrast, the plasma concentration of preβ1-HDL-C fraction, seemed to be an independent determinant of CKD severity. On the other hand, in a larger cohort study with non-dialyzed patients at stages 2–4 of CKD [45], it was noted that both circulating PON1 protein levels and lactonase activity were significantly lower than NC across all stages of CKD. This was in agreement with other reports regarding PON1 paraoxonase and arylesterase activities [38,40] and PON1 concentration [46] in non-dialyzed patients. Interestingly, circulating PON lactonase, but not protein levels, predicted higher risk of future adverse clinical outcomes in patients with CKD, even after adjusting for traditional risk factors [45]. Nevertheless, larger sample-sized researches are needed to verify those findings.

## 3. Main factors influencing PON1 activity in chronic kidney disease

### 3.1. Effect of genetic polymorphism

The PON1 gene cluster maps to human chromosome 7q21–22. Several polymorphisms in the promoter and coding regions affect gene expression levels and PON1 activity [3,47,48]. The most common polymorphisms in the PON1-coding region are L55M, including a leucine (L) to methionine (M) substitution at codon 55, and Q192R including a glutamine (Q) to arginine (R) substitution at codon 192. The PON1-promoter region contains five polymorphisms [C (–108) T, C (–126) G, A (–162) G, C (–907) G and A (–1074) G], with polymorphism C (–107) T having the greatest impact. The 55 L allele results in significantly higher mRNA and protein levels and therefore higher activity compared to 55 M allele [3]. The −108 C allele has greater promoter activity than the −108 T allele, which leads to a higher serum activity. Regarding the Q192R polymorphism, arginine at position 192 specifies high PON activity, whereas a glutamine specifies the low activity variant [49].

Previous reports have shown that paraoxonase Q192R gene polymorphism is an independent cardiovascular risk factor in non-insulin-dependent diabetic patients [50,51]. In this context, there has been increasing interest in determining whether polymorphisms of PON1 gene also contribute to the development of diabetic nephropathy (DN). It was found that Q192R was associated with the prevalence of nephropathy together with retinopathy in type 2 diabetes mellitus (T2DM) patients, whereas no associations were observed between microangiopathies and the other polymorphisms examined [52]. In T2DM patients on HD, only C (–108) T (or else rs705379) polymorphism was associated with nephropathy in the recessive and additive inheritance modes [53]. Additionally, T2DM patients with nephropathy bearing the L55 (or else rs854560 T) allele were at higher risk for ischemic cerebral stroke. PON1 polymorphisms were also studied in another Caucasian population with type 1 diabetes mellitus (T1DM), where it was found that genotypes LL on 55, AA (–162) and GG (–1074), were associated with an increased urinary albumin loss [54]. However, inconsistent with the above findings were the results obtained in other ethnic groups, in which PON1 L55M and Q192R polymorphisms [55,56] as well as C (–108) T [57], were not associated with CKD in T2D nor in T1D.

In CKD patients on HD, the most frequent genotype was heterozygosity for L55M polymorphism, which was associated to higher PON1 activity than MM, and lower than the LL genotype [58]. QQ was the most frequent genotype for Q192R polymorphisms, presenting the lowest PON1 activity, as compared to QR and RR genotypes. Multiple regression analysis identified heterozygosity and homozygosity for L55M and Q192R polymorphisms (together with LDL-C, Apo-A1, time of dialysis and CRP levels), as independent variables associated with PON1 activity. Furthermore, in CKD patients, the R allele of the Q192R variant was found to be associated with oxidative stress as assessed by plasma 8-isoprostanes, and was dose-dependently related to the severity of left ventricular hypertrophy and left ventricular systolic dysfunction [59]. On the other hand, there is evidence in the literature excluding the association between the PON1 gene variants, conferring higher enzyme activity, and the increased cardiovascular risk in renal transplant recipients [60]. Furthermore, in elderly patients with mild CKD, it has been proved that PON1 Q192 and L55M gene polymorphism may not constitute regulatory factor in the progression of renal failure [61]. However, larger- sized, longitudinal studies, focusing on long-term follow-up, taking into account patient medications are needed to confirm the above findings.

### 3.2. Association with the lipid profile

It is known that the association of PON1 with HDL is indispensable for preserving normal serum PON1 activity, as HDL assures an amphipathic environment which, not only protects the hydrophobic N-terminal site of the enzyme, but also facilitates the interaction of PON1 with its substrates [4]. In patients with CKD, changes in serum lipid profile (notably, significantly lower HDL-C and Apo-AI levels), seemed to be related with a decreased PON1 serum activity [58,62,63]. Furthermore, it has been proved that HDL from HD and PD patients shifted toward a more proinflammatory phenotype, with remarkable alterations in the lipid moiety (lower levels of cholesteryl ester and phospholipids, and higher levels of triglycerides) and in the protein composition (lower amounts of Apo A-I, Apo AII and paraoxonase 1) [64]. These alterations reflected on a substantially impaired capability of HDL to promote cholesterol efflux from macrophages. The dysfunctionality of HDL was accompanied by a decrease in arylesterase activity of HDL-associated paraoxonase, in both HD and PD patients. Additionally, it has been shown that changes in the distribution of HDL2 and HDL3 subclasses of HDL in patients on HD, may contribute to the decrease in PON1 activity [65]. The reduction in the concentration of HDL3 subfraction of HDL, which was found to carry 95% of serum PON1 activity, was suggested the most important determinant of PON1 activity decrease in HD.

In a larger-scaled report, the genotypes for several polymorphisms of various candidate genes for CKD were determined [66]. It was demonstrated that among the seven different polymorphisms found to be significantly associated with the prevalence of CKD in individuals with low or high serum concentrations of triglycerides, HDL or LDL, the Q192R polymorphism of PON1, known to influence PON1 activity levels [58,59], was detected in individuals with high serum LDL-C concentration. Although assumptions about PON1 activity are not supported by this study, the above findings indirectly infer the significance of lipid profile and LDL-C levels to PON1 activity of CKD patients.

### 3.3. Association with oxidative stress

In patients with CKD, the increased production of reaction oxygen species, resulting from inflammation and dialysis, the decreased clearance of pro-oxidant substances due to renal dysfunction, as well as the attenuation of antioxidant defense system, lead to increased oxidative stress [67,68]. PON1 activity, representing a substantial enzymatic activity related to HDL, was studied in many reports either as a contributor to plasma antioxidant mechanisms [62,69,70,87] or as an important antioxidant factor [34,39,40,71]. It was found that the significant decrease in PON1 activity in CKD patients was correlated with an increase in the serum levels of malonaldehyde (MDA), a finding that was ascribed to the imbalance between lipid peroxidation and antioxidant system [62]. In patients on HD, an inverse correlation between paraoxonase/arylesterase activities and serum lipid hydroperoxides (LOOH) levels was also reported [39]. The comparison of subjects with varying degrees of renal insufficiency, some of who were undergoing HD or PD, showed that patients on HD with greatest magnitude of renal impairment, had a corresponding significant reduction in antioxidant activity, as indicated by decreased paraoxonase and glutathione peroxidase (GSH-Px) activities [70]. Moreover, paraoxonase activity levels in those patients were negatively correlated with the serum levels of F2-isoprostanes, the prostaglandin-like products linked to lipid peroxidation of arachidonic acid and oxidative damage.

The relationship between the HDL-associated PON1 activity and the plasma concentration of protein carbonyls, which are products of protein oxidation and also pathogenic mediators of cardiovascular complications, were analyzed in another cross-sectional study with ESRD patients on HD, and type I diabetes subjects without complications [71]. The data revealed that in ESRD patients, protein carbonyl concentration was significantly higher than in diabetics and controls, and was inversely related with PON1 activity. Interestingly, in diabetic patients the impaired efficacy of removal of lipid peroxides (LPO) by HDL was found to be associated to glycoxidation rather than PON1 activity.

### 3.4. The role of carbamylation

Carbamylation is a post-translational modification of proteins or free amino acids, frequently seen in uremic patients. It occurs either spontaneously, when amino acid residues react with urea decomposition products (such as isocyanate), or by the myeloperoxidase (MPO)-catalyzed pathway [72]. There is evidence revealing that circulating HDL can be carbamylated as well, especially in inflammatory conditions like type 2 diabetes, CAD and CKD [73]. In CKD patients, HDL carbamylation was shown to play a crucial role in the development and progression of atherosclerotic cardiovascular disease. Recent data demonstrated that the carbamylation level of HDL in ESRD patients was increased compared to healthy individuals, and was correlated to the decrease in HDL- PON1 activity [74]. Furthermore, carbamylated HDL *in vitro* inhibited endothelial cell repair functions *via* downregulation of VEGF receptor 2 pathway.

Contemporary observations ascertained the carbamylation of PON1 in uremic HDL [75]. The degree of PON1 carbamylation in HDL isolated from patients on HD was higher than in control HDL. The nanoLC-MS/MS technique proved that the carbamylation of lysine 290 (K290) of PON1, a residue adjacent to PON1 activity determining site, was identified solely in uremic HDL and not in control HDL. This K290 carbamylation resulted in local conformational changes, which reduced surface area and affected tertiary PON1 structure. It was thus conceivable that carbamylation of PON1 in uremic patients could be one of the factors impairing PON1 activity and HDL antioxidant function.

### 3.5. Effect of drugs, antioxidant therapy and nutritional status

There is considerable evidence in the literature concerning the effect of drugs on PON1 activity in patients with CKD. It has been shown that erythropoietin β and iron treatment has a beneficial effect on the levels of antibodies against oxidized LDL and on serum PON1 activity in pre dialysis patients with chronic renal disease and anemia [76]. Immunosuppressive drugs and especially the calcineurin inhibitor tacrolimus, may be more effective than cyclosporine as regards paraoxonase and arylesterase activities in CKD patients before and after 12 months of kidney transplant (KT) [77]. The modulating effect of hydroxymethyl glutaryl-coenzyme A (HMG-CoA) inhibitors (statins) on PON1 activity in type 2 diabetic nephropathy (T2DN) patients was investigated as well [68–70]. It was observed that diabetic patients with or without nephropathy, who received atorvastatin for 12 weeks, had significantly higher levels of PON1 activity at the end of the treatment than before treatment [78]. PON1 activity was positively correlated with serum HDL-C levels and negatively with LDL-C and glucose levels in all patients. The upregulation of PON1 by atorvastatin could be explained by means of the *in vitro* results from human hepatoma HepG2 cells and human embryonic kidney 293 cells, which revealed that atorvastatin, as simvastatin and pitavastatin, increase PON1 promoter activity through an inhibition of mevalonic acid-derived famesyl pyrophosphate (FFP) pathway [79]. On the other hand, it was proved that administration of lovastatin to patients with T2DN for 90 days produced a significant improvement in PON and arylesterase activities and LDL-C lag phase [80]. Nevertheless, in contrast to the atorvastatin study (68), the increase in PON1 activity under the influence of lovastatin therapy appeared to be independent of HDL-C level and was assumed to be influenced by statin’s antioxidant properties.

The efficacy of antioxidant compounds on the enhancement of PON1 activity in CKD patients was analyzed in several studies [81–85]. It has been shown that zinc supplementation for 2 months in HD patients resulted in a significant increase in PON1 activity compared to non-treated HD patients [81]. Daily ingestion of purified extract of pomegranate polyphenols in HD patients produced a significant increase in PON1 activity compared to the placebo group, without having any effect on other markers of CVD risk, inflammation or oxidative stress [82]. Similarly, intravenous administration of vitamin C in HD patients for 6 months exerted a beneficial effect, with an increase in antioxidant defense evaluated by PON1 activity, and a decrease in oxidative stress markers (lipid hydroperoxides and AGE adducts) compared to non-supplemented HD patients [83]. This was in line with *in vitro* preliminary findings showing that vitamin C protects PON1 activity from oxidative damage triggered by lipophilic peroxyl radical initiators [84]. Nevertheless, there is also evidence suggesting that treatment of HD patients with a potent antioxidant cocktail containing high doses of the antioxidant vitamins C, E, B_6_, B_12_ and folic acid for 8 weeks, failed to improve PON1, catalase and GPX activities [85].

The effect of nutritional status on PON1 activity was also studied in CKD patients. It was noted that there was no significant difference in PON1 paraoxonase and lactonase activities between malnourished (BMI < 20 kg/m^2^), normal weighted and obese (BMI > 30 kg/m^2^) HD patients [86]. Multiple regression analysis showed that PON1 lactonase activity, CRP levels and leptin concentration were independent predictors of paraoxonase activity [86]. The impact of PON1 activity levels along with inflammatory parameters (CRP and IL-6) and nutritional status (estimated through serum albumin and BMI), were assessed on HD patients’ survival during a 3-year period [87]. The data suggested that lower PON1 activities in combination with malnutrition and inflammation contributed to higher mortality in patients on long-term HD.

## 4. PON1 activity and cardiovascular risk in chronic kidney disease

It is well-established that CKD patients are among populations at higher risk for cardiovascular (CV) disease and that this is partly ascribed to enhanced oxidative stress [68,88]. Reduced serum magnesium, closely related to the risk of developing vascular calcification and premature vascular aging, also contributes to increased oxidative stress and chronic inflammation [89]. Oxidative stress, intensified in uremia by increased production of reactive oxygen species (ROS) and attenuated clearance of pro-oxidant substances, together with diminished antioxidant defenses, confers to the modification of circulating lipids and lipoproteins. In patients with coronary artery disease (CAD), PON1 was found to play a prominent protective role in the development of atherosclerosis, and the decrease of its activity in serum predicted the degree of coronary lesion [90,91]. Regarding the CKD patients, there are essential data demonstrating the association of PON1 activity with cardiovascular alterations, as described below.

The relation of paraoxonase activity with vascular disease in CKD was initially investigated in CRF patients on HD and in non-dialyzed CRF patients, by assessment of common carotid artery intima-media thickness (IMT), a useful marker for noninvasive atherosclerosis imaging [92]. It was proved that IMT in HD patients correlated inversely with the basal and salt-stimulated PON1 activity level, although it did not correlate with HDL and LDL serum concentrations or basal PON1/HDL ratio. In addition, it was demonstrated that the most significant determinant of IMT in HD and CRF patients was arylesterase activity of PON1, followed by CRP serum levels (Table 1). Likewise, in renal transplant recipients (RT), PON1 activity was negatively correlated with systolic and diastolic blood pressure, with mean arterial pressure and carotid-femoral pulse wave velocity (cf-PWV) [93]. In a model of linear regression including age, gender, diabetes, mean arterial pressure, urine protein level and creatinine clearance, PON1 was a predictor of cf-PWV. Therefore, it was concluded that reduced PON1 activity in RT recipients is significantly associated with arterial stiffening, a fact that was attributed to increased oxidized LDL, which stimulates collagen synthesis in atrial smooth muscle and causes intimal thickening. Furthermore, the association of PON1 activity with epicardial fat tissue (EFT) was analyzed in RT and HD patients [94,95]. The enlargement of EFT, observed in many pathological conditions connected with increased ROS production like CKD, is considered as another cardiovascular risk factor [96]. It was found that PON1 activity was negatively correlated with EFT in RT patients [94] and in ESRD individuals on HD [95]. In a multivariate regression analysis model containing several independent variables, it was found that PON1 activity plus age, BMI, total cholesterol, HDL-C and triglycerides levels were independent predictors of EFT [95].

**Table 1.** Association of PON1 activity with risk factors for cardiovascular disease in patients with CKD.

Reference	Biomarker	Association with PON1	Detection method	Levels In CKD	Implications
Saeed et al. [92]	Carotid IMT	Basal PON1 HD: *r* = –0.46, *p* = 0.01 CRF: *r* = –0.70, *p* < 0.001 Arylesterase HD: *r* = –0.70, *p* < 0.001 CRF: *r* = –0.75, *p* < 0.001	Paraoxonase assay (- /+ NaCl stimulation) Arylesterase assay (phenyl acetate) in clinical chemistry analyzer	Basal PON1 HD: 133 ± 19 U/L CRF:142 ± 22 U/L NC:180 ± 28 U/L Arylesterase HD: 77 ± 13 U/L CRF:78 ± 18 U/L NC:103 ± 15 U/L	Iin both HD and CRF patients, the most significant determinant of carotid IMT was PON1 arylesterase activity.
Gungor et al. [93]	Systolic and diastolic BP, MAP, cf-PWV	Systolic and diastolic BP: *r* = –0.287, *p* = 0.01 Diastolic BP: *r* = –0.371, *p* = 0.002 MAP: *r* = –0.345, *p* = 0.05 cf-PWV: *r* = –0.274, *p* = 0.02)	Paraoxonase assay in clinical chemistry analyzer	75 ± 52 U/L	Reduced PON1 activity was significantly associated with increased arterial stiffness in RT patients.
Abdallah et al. [95]	Epicardial adipose tissue thickness (EATT)	*r* = –0.484, *p* < 0.0001	Paraoxonase assay kit	HD:82 ± 32UL NC: 164 ± 62 U/L	PON1 activity inversely correlated with EATT and was one of the independent predictors of EATT in HD patients.

Data of activity are expressed as mean ± SD. HD: hemodialysis; CRF: chronic renal failure; IMT: intima-media thickness; RT: renal transplant; BP: blood pressure; MAP: mean arterial pressure; cf-PWV: carotid- femoral pulse wave velocity; CKD: chronic kidney disease; LVMI: left ventricular mass index; LVEF: left ventricular ejection fraction; LVH: left ventricular hypertrophy; LV: left ventricular; T1DM: type 1 diabetes mellitus.

The possible associations of PON1 lactonase activity and circulating fibroblast growth factor 23 (FGF-23) levels with the incidence of native arteriovenous (AV) fistula thrombosis were examined in chronic HD patients, as AV thrombosis accounts for a great percentage of vascular access loss, developing in venous segment of native AV fistula or AV graft [97]. The results demonstrated that PON1 lactonase activity was significantly decreased, whereas FGF-23 was significantly elevated in HD patients with thrombosed AV fistula compared with those with non-thrombosed AV fistula. The significant negative correlation between PON1 lactonase and FGF-23, which represents a central regulator of vascular dysfunction in CKD [98], may support the suggestion that FGF-23 and PON1 lactonase values may contribute to the prediction of native AV fistula thrombosis in HD patients [97]. In a longitudinal study with CKD patients followed- up for 3 years, it was shown that the PON1 Q192R gene variant is implicated in left ventricular (LV) hypertrophy and in LV dysfunction [59], which are surrogate indicators of CVD. The R allele of the Q192R variant in the PON1 gene, was dose-dependently related in a direct fashion to LV mass index and in an inverse fashion to LV ejection fraction. Multiple regression analysis with adjustment for HDL and eGFR, did not modify those associations. Consequently, despite the lack of direct data of PON1 activity measurement, it was hypothesized that the prooxidant state generated in CKD patients by low paraoxonase activity linked with the R allele of Q192R variant, may be related to the longitudinal evolution of LV dysfunction. On the other hand, the study of the relationship between PON1 lactonase activity and a number of CV risk factors, such as homocysteine, cystatin C and ADMA levels in HD and RT patients, showed that paraoxonase activity and homocysteine level were independent predictors of lactonase activity [99]. When PON arylesterase and Apo A-I were investigated in relation with the mortality risk in maintenance HD patients, it was found that high PON activity was associated with lower risk of 12-month mortality, whereas there was no difference of mortality risk across HDL-C levels [100]. In addition, the combination of high PON activity and low Apo A-I, compared with low PON and low Apo A-I was connected with lower mortality risk in patients. In a larger scaled population, consisting of participants in the Prevention of Renal and Vascular End-stage Disease (PREVENT) study, followed-up during 9.3 years with recording CVD events, the results confirmed that PON1 activity correlated with several cardiovascular risk markers, and more strongly with HDL-C and Apo A-I [101]. After adjusting for conventional risk factors, an approximately log-linear association of PON1 with risk of CVD was determined, which, however, was attenuated on further adjustment for HDL-C. This may indicate that the log-linear inverse association between PON1 activity and CVD risk is partly dependent on HDL-C levels. A meta-analysis of available published prospective cohorts provided evidence that adding PON1 activity to a prediction model containing conventional risk factors did not improve index of risk prediction. However, certain limitations were stated in this study, such as the possibility of subclinical or undetected prevalent disease, the comparison of two types of PON1 activity, arylesterase and paraoxonase in the PREVENT and meta-analytic parts of the study and the prolonged serum storage of samples before analysis.

In view of the fact that albuminuria in type 1 diabetes mellitus (T1DM) increases the risk of progressing to ESRD, alterations in the HDL proteome from T1DM patients were analyzed in relation to albuminuria and CVD [102]. By means of isotope dilution tandem-mass spectrometry to quantify various proteins in HDL and by coronary artery calcification (CAC) measurements to assess the prevalence of CVD, it was demonstrated that among HDL proteins altered in T1DM subjects, only PON1 and PON3 were associated with the presence of CAC. The mean levels of PON1 and PON3 in HDL isolated from subjects with CAC were significantly lower than those from subjects without CAC. It is interesting though that only one protein, PON1, was associated strongly and negatively with both albumin excretion and CAC, and that PON1 mass was positively correlated with PON1 activity. Hence, the strength of the study is that in the HDL proteome which was found remodeled in T1DM patients with albuminuria, only PON1 seemed to be associated with both albuminuria and CVD. Nevertheless, there were study restrictions mentioned, for instance the small number of female subjects with albuminuria and certainly, the cross-sectional design, which could not reveal whether the association of PON1 with CAC and albuminuria was causal.

Lately, the impact of PON1 deficiency on the development of oxidative stress and detrimental clinical outcome in CKD was evaluated *via* well-characterized animal model of high-salt induced renal disease [103]. By comparing control Dahl salt-sensitive rats (SS-WT rats) with mutant PON1 knockout rats (SS-PON1 KO rats), maintained both on high salts diets for five weeks to induce hypertensive renal disease, it was observed that SS-PON1 KO rats developed compensated left ventricular hypertrophy after only 4 weeks on high salts diet. Upon examining the kidneys, SS-PON1 KO demonstrated increased recruitment of CD68 positive immune cells in renal interstitial, as well as increased expression of the key inflammatory genes (Timp-1, MCP-1, IL-6, COL1A1 and TGFβ) compared to the SS rats. This supported the suggestion that PON1 suppresses macrophage pro-inflammatory responses and decreases sustained proinflammatory reactions. Moreover, a significant increase in the expression of genes linked to cardiac hypertrophy, as well as a significant decrease in genes linked to left ventricular function, were apparent in SS-PON1 KO rats. Those findings underlined the importance of PON1 as anti-atherogenic enzyme, with cardioprotective functions. It was also reported that PON1 may serve as a potential therapeutic target to help reduce cardiac morbidity and mortality in high-risk CKD population.

## 5. Evaluation of PON1 activity as a prognostic and diagnostic tool in chronic kidney disease

**Figure 2.**
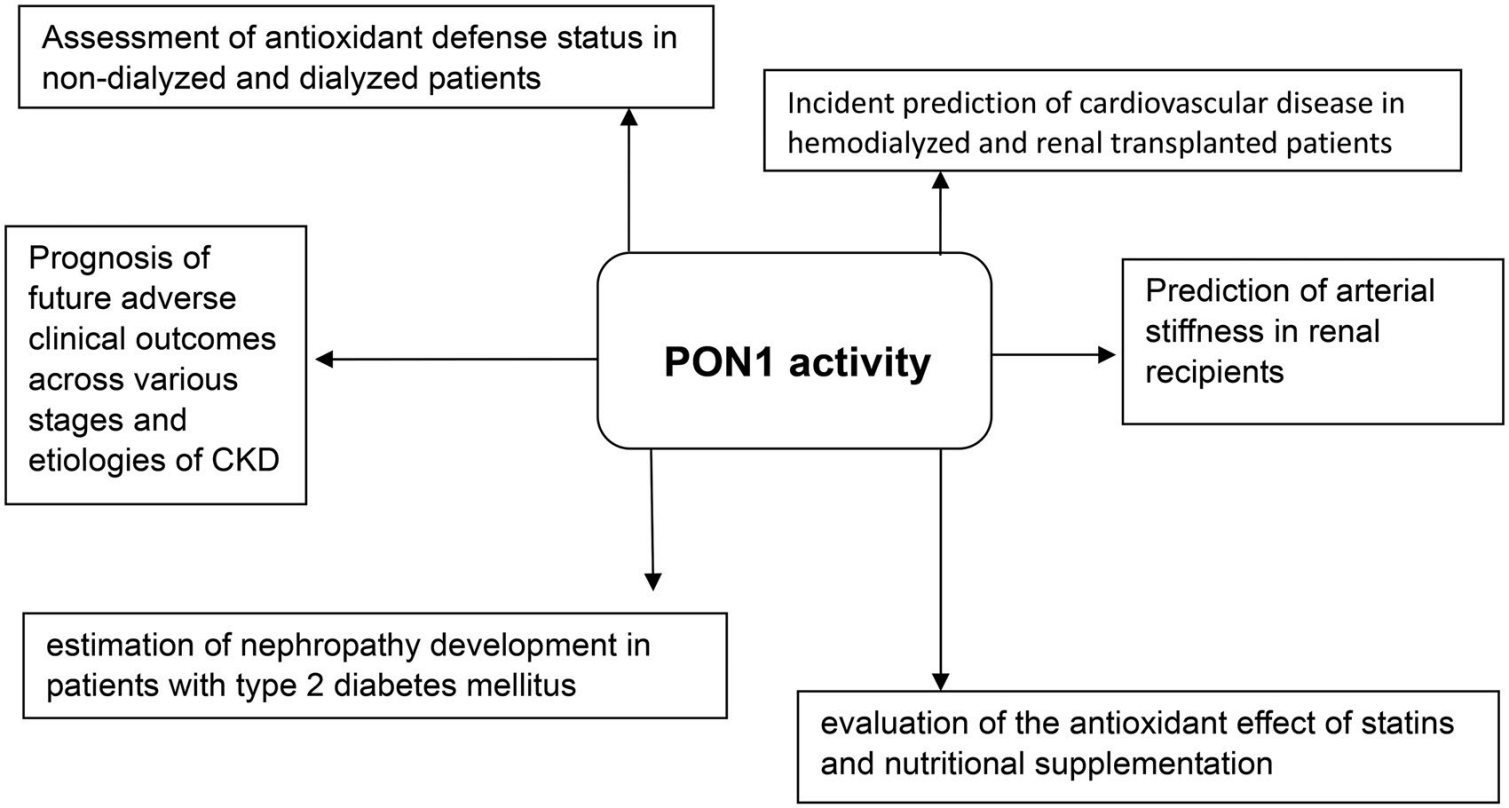
The importance of PON1 activity measurement in chronic kidney disease (CKD).

There are multiple lines of evidence in the literature emphasizing or suggesting the importance of PON1 activity in kidney diseases. In the first place, PON1 activity has been largely used to estimate the antioxidant defense status in patients at various stages of CKD or on different dialysis treatments [31,104–108] (Figure 2). In this context, the salutary effect of hemodialysis on HDL anti-inflammatory activity of patients with ESRD [35,109], but also the efficacy of non-pharmacological methods, such as resistance training models applied in CKD patients not needing HD [110], were determined by means of PON1 activity. In addition, based on the antiatherogenic properties of PON1 and its ability to quell LDL susceptibility to oxidation, numerous studies indicated that the evaluation of PON1 activity represents a helpful approach to monitor pharmacological and nutritional antioxidant treatments in CKD [13,78,80,82,83,111].

On the other hand, PON1 lactonase activity playing protective role against homocysteinylation, has been proposed to constitute a potential new predictor of cardiovascular risk in both hemodialyzed and renal transplanted patients [99,112]. Diminished circulating PON1 lactonase activity and decreased serum arylesterase activity could have prognostic utility on future adverse clinical outcomes in CKD patients [33,113]. In patients on maintenance hemodialysis, low serum PON1 activity was documented to correlate with high risk of 12-month all-cause mortality [100], whereas in renal transplant recipients, it was suggested to contribute to the prediction of arterial stiffness [93]. However, there was data which revealed that paraoxonase activity could not independently predict cardiovascular outcome among non-dialysis CKD patients [114], and that the inverse association between PON1 activity and CVD risk was partly dependent on HDL-C levels [101], possibly suggesting that PON1 activity may have an auxiliary role for CVD risk assessment.

In type 2 diabetes mellitus (T2DM), decreased PON1 activity was found to associate with enhanced oxidative stress in patients with diabetic nephropathy [23,24,115], along with the development of vascular complications [14,18]. Additionally, PON1 activity levels on HDL derived from T2D patients with microalbuminuria or macroalbuminuria, and HDL antioxidative ability in protecting LDL from oxidation were significantly decreased compared with those from normoalbuminuric patients and healthy individuals [116]. Thus, decreased levels of HDL-PON1 activity in patients with T2DM are plausibly indicative of incipient or overt nephropathy. Importantly, considering the advantages of measurement of arylesterase over paraoxonase activity, which has lower inter-individual variability and is minimally affected by PON1 polymorphisms, there were studies proposing arylestease and not paraoxonase PON1 as a biomarker for predicting the progression of CKD in T2DM [117,118].

It is interesting to note that PON1 was further used to assay drug-induced nephrotoxicity in animal models; in order to determine the effect of natural antioxidants, such as coenzyme Q10 against gentamycin-induced kidney injury [119], but also the potential of anti-inflammatory encapsuled materials, such as itaconyl chondroitin sulfate nanogel in combating chronic kidney disorders induced by the cytotoxic cancer medicines cisplatin and doxorubicin [120]. In both cases, the beneficial effect of the above antioxidative factors was estimated by means of PON1 activity measurement, which additionally was found to be negatively correlated with atherogenic index in the animals treated with coenzyme Q10 [119].

Overall, it is clear that PON1 activity has prognostic and diagnostic value in CKD, as lower values would indicate impairment of antioxidant defense as well as oxidant-antioxidant imbalance involved in the etiology of cardiovascular risk. Therefore, the increase of PON1 expression and activity by nutritional and pharmacological means. may represent a potential therapeutic target for treating chronic kidney disease and attenuating renal disease progression. Nevertheless, it appears that large-scaled, multicentered clinical trials pend to ascertain the applicability of PON1 activity as a biomarker. Also, the great heterogeneity in the selection of the substrates and the conditions of the assay used with each substrate, possibly lead to differences in the comparison of values and the interpretation of the results [121]. For example, according to meta-analysis data, when paraoxonase used as substrate, a gradient reduction of PON1 activity along with the worsening of CKD stages became apparent. This phenomenon showed the same trend, but was not significant when arylesterase was determined [106]. Consequently, in anticipation of standardization of PON1 activity assays, sources of laboratory variability (e.g., storage duration of samples, freeze-thaw, storage temperature), should be monitored through the research analyses [122]. Quality control procedures are also strongly recommended, especially when longitudinal studies are conducted.

## 6. Conclusions and future perspectives

PON1 constitutes a significant component on HDL, capable of hydrolyzing a variety of substrates. Due to its different catalytic sites and structural conformations, it is responsible for various activities and it is deemed to play important antioxidative and anti-inflammatory roles in atherosclerosis-related diseases, such as chronic kidney disease. There is emerging data to suggest that PON1 activity has clinical relevance. In pre-dialysis, dialysis and renal transplant patients, PON1 activity measurement may contribute to the evaluation of antioxidant status. Additionally, it may predict future adverse clinical outcomes, as well as possible risk for cardiovascular disease. In patients with type 2 mellitus, PON1 activity could represent an adjunctive indicator of the progression of CKD. However, the generalization of these results awaits confirmation in larger CKD cohorts.

Future perspectives should address to a deep understanding of the translational regulation of PON1, which could enhance the efficacy of pharmacological and nutritional strategies for increasing PON1 activity levels and preventing or reducing cardiovascular complications. Cohort or cross-sectional studies exploring the role of PON1 in hypertensive nephropathy or in IgA nephropathy in relation to inflammation, where literature evidence is limited, are also required. Furthermore, the standardization of PON1 activity methods, coupled with the identification of the natural substrates of PON1, are also indispensable, so as to define better the importance of PON1 as biomarker in chronic kidney disease.

## Supplementary Material

PON1 Legends to Figures revided.doc

PON1 Figure 2 revised.doc

PON1 Figure 1 revised.doc

## Data Availability

The data used to support this article will be provided upon request from the corresponding author.
