# Supplementary material for: The importance of paraoxonase 1 activity in chronic kidney disease
Source: Ren Fail. 2024 Jul 10;46(2):2376930. doi: 10.1080/0886022X.2024.2376930 (PMC11238655; doi:10.1080/0886022X.2024.2376930)
Supplement: PON1 Legends to Figures revided.doc [file IRNF_A_2376930_SM6204.doc]

Figure 1

Potential causes of reduced PON1 activity levels in chronic kidney disease (CKD).

Figure 2.

The importance of PON1 activity measurement in chronic kidney disease (CKD)
