# Supplementary material for: The importance of paraoxonase 1 activity in chronic kidney disease
Source: Ren Fail. 2024 Jul 10;46(2):2376930. doi: 10.1080/0886022X.2024.2376930 (PMC11238655; doi:10.1080/0886022X.2024.2376930)
Supplement: PON1 Figure 2 revised.doc [file IRNF_A_2376930_SM6203.doc]

Assessment of antioxidant defense status in non-dialyzed and dialyzed patients

status in non-dialyzed and dialyzed patients

status in non-dialyzed and dialyzed patients

Incident prediction of cardiovascular disease in hemodialyzed and renal transplanted patients

**PON1 activity**

Figure 2.

The importance of PON1 activity measurement in chronic kidney disease (CKD)

Prediction of arterial stiffness in renal recipients

Prognosis of future adverse clinical outcomes across various stages and etiologies of CKD

estimation of nephropathy development in patients with type 2 diabetes mellitus

evaluation of the antioxidant effect of statins and nutritional supplementation
