# Supplementary material for: The importance of paraoxonase 1 activity in chronic kidney disease
Source: Ren Fail. 2024 Jul 10;46(2):2376930. doi: 10.1080/0886022X.2024.2376930 (PMC11238655; doi:10.1080/0886022X.2024.2376930)
Supplement: PON1 Figure 1 revised.doc [file IRNF_A_2376930_SM6202.doc]

HDL-C and apo A-I concentrations

Oxidized lipids

Altered HDL-C subclasses distribution

PON1 concentration

Clearance of AGE adducts

PON1 carbamylation

Phenotype distribution

Figure 1

Potential causes of reduced PON1 activity levels in chronic kidney disease (CKD).
